# The relationship between mood state and perceived control in contingency learning: effects of individualist and collectivist values

**DOI:** 10.3389/fpsyg.2015.01430

**Published:** 2015-09-29

**Authors:** Rachel M. Msetfi, Diana E. Kornbrot, Helena Matute, Robin A. Murphy

**Affiliations:** ^1^Department of Psychology, Centre for Social Issues Research, University of LimerickLimerick, Ireland; ^2^Health Research Institute, University of LimerickLimerick, Ireland; ^3^Department of Psychology and Sport, University of HertfordshireHatfield, UK; ^4^Faculty of Psychology and Education, University of DeustoBilbao, Spain; ^5^Department of Experimental Psychology, University of OxfordOxford, UK

**Keywords:** perceived control, contingency judgment, depression, bipolar disorder, illusion of control, outcome density, individualism, collectivism

## Abstract

Perceived control in contingency learning is linked to psychological wellbeing with low levels of perceived control thought to be a cause or consequence of depression and high levels of control considered to be the hallmark of mental healthiness. However, it is not clear whether this is a universal phenomenon or whether the value that people ascribe to control influences these relationships. Here we hypothesize that values affect learning about control contingencies and influence the relationship between perceived control and symptoms of mood disorders. We tested these hypotheses with European university samples who were categorized as endorsing (or not) values relevant to control—individualist and collectivist values. Three online experimental contingency learning studies (*N*_1_ = 127, *N*_2_ = 324, *N*_3_ = 272) were carried out. Evidence suggested that individualist values influenced basic learning processes via an effect on learning about the context in which events took place. Participants who endorsed individualist values made control judgments that were more in line with an elemental associative learning model, whilst those who were ambivalent about individualist values made judgments that were more consistent with a configural process. High levels of perceived control and individualist values were directly associated with increased euphoric symptoms of bipolar disorder, and such values completely mediated the relation between perceived control and symptoms. The effect of low perceived control on depression was moderated by collectivist values. Anxiety created by dissonance between values and task may be a catalyst for developing mood symptoms. Conclusions are that values play a significant intermediary role in the relation between perceived control and symptoms of mood disturbance.

## Introduction

People who feel “in control” of their lives tend to be healthier than those who feel that they are helpless to affect change (e.g., Seligman, 1975; Taylor and Brown, 1988). Whilst this statement is sometimes accepted without question, there is good reason to question its veracity (e.g., Yamaguchi et al., 2008; Cheng et al., 2013) because people's differing values shape the interpretation of their experiences (Markus and Kitayama, 1991; Triandis, 1996; Green et al., 2005). It is therefore important to understand how values intervene in the relation between perceived control and symptoms of psychopathology. Here we report on a series of three experiments that were carried out to this end. First, we describe robust methods of studying perceived control, evidence of judgment bias and links to symptomatology, before discussing the intervening role of values.

### Perceived control and contingency judgments

Different methods have been used to study perceived control and the relation to mental health (e.g., contingency judgements, Alloy and Abramson, 1979; predictions of random events, Fast et al., 2009; chance and skill tasks, Langer, 1975) in order to address whether people's judgments are an accurate reflection of their experiences. However, in order to determine accuracy, the method used must provide an objective measure of experienced control. Contingency judgment methods provide such a measure (Dobson and Franche, 1989).

In a standard contingency task, participants are asked to perform actions that may or may not result in an outcome (e.g., button press, light flash). After numerous such experiences, participants rate their own control over the outcome using a numeric scale (e.g., +100 complete control, 0 no control, −100 completely prevent). The experimenter can program the contingency between action and outcome, which can be defined by the four possible action-outcome conjunctions shown in Table 1, and compare it to participants' ratings for accuracy.

**Table 1:** **Examples of the frequencies of stimulus conjunctions, including action, outcome and context, which can be used to calculate ΔP in conditions with no control and a low (0.25|0.25) and high (0.75|0.75) density of outcomes**.

**Stimuli present in the learning scenario**			**Action—outcome conjunctions**	**Condition**	
**Action**	**Outcome**	**Context**		**0.25 | 0.25**	**0.75 | 0.75**
+	+	+	A	5	15
+	−	+	B	15	5
−	+	+	C	5	15
−	−	+	D	15	5
*p*(outcome|action) = *p*(O|A)			A/A+B	0.25	0.75
*p*(outcome|no action) = *p*(O|~A)			C/C+D	0.25	0.75
Outcome density = *p*(outcome)			(A+C)/(A+B+C+D)	0.25	0.75
Δ*P* = *p* (O|A) −*p*(O|~A)			(A/(A+B)) − (C/(C+D))	0	0

*^+^Indicates the occurrence of a stimulus, whereas ^−^indicates its absence*.

ΔP (delta P) is the most appropriate measure of the contingency between binary events (Allan, 1980) and is calculated as ΔP = *p*(outcome|action) - *p*(outcome|no action). ΔP is a value that varies between +1 (a perfect positive contingency, complete control), through zero (no control) to −1 (actions prevent the outcome occurring). This scale, like the judgment scale described above, is continuous with opposite upper and lower bounds (−100 and +100 for judgment scales, and −1 and +1 for ΔP). Note that the experimental setting or context can also be thought of as a stimulus and reference to this is included in Table 1. This is because in order to assess their control, people must also attend to events that are outside their control and occur randomly in the same context. Thus experimental studies often require that participants rate their own control over the outcome and that of the context so this aspect of learning can be studied (e.g., Msetfi et al., 2013).

### Judgment bias and wellbeing

Judgments of control are sensitive to quite subtle changes in ΔP and people tend to recognize when their actions do and do not have control over outcomes (e.g., Wasserman et al., 1993). A detailed review of these studies is outside the scope of this paper. However, several key findings are particularly relevant. The first finding is that when participants are exposed to conditions like those in Table 1, which have the same zero level of contingency but a different density of outcomes occurring over time, judgments tend to be higher when outcomes occur frequently (0.75|0.75 condition). With infrequently occurring outcomes, judgments are closer to zero and more consistent with ΔP (e.g., Allan and Jenkins, 1980, 1983; Dickinson et al., 1984). This difference in judgments between the two conditions is known as the “outcome density effect” (for a discussion see Allan, 1993) but is also referred to as the “illusion of control” (e.g., Alloy et al., 1981; Matute, 1996; Blanco et al., 2011). The illusion of control refers to the observation that while the objective contingency is zero, participants feel that they have at least a moderate degree of control over the outcome. This finding has been influential in terms of theoretical developments in the field of human contingency judgments, leading to the search for the “best” rule to describe such systematic departures from the normative ΔP, though often leading to the conclusion that simple associative processes provide the best account (Allan, 1993).

The second key finding to note here is that illusory control or outcome density effects are not evident for people with mild depression. For example, Alloy and Abramson (1979), and others, presented non-depressed and mildly depressed participants with the conditions described in Table 1. Non-depressed participants showed the outcome density effect, whereas those who were mildly depressed did not. In fact, overall, their control judgments were closer to the objective contingency programmed by the experimenter. This finding was labeled “depressive realism” with the implication that the depressed recognized their lack of control because it was consistent with motivational and negative bias aspects of depression (Ackermann and DeRubeis, 1991; Haaga and Beck, 1995; Moore and Fresco, 2012). Thus, illusory control was conceived as evidence of healthy biases and optimism, serving as a protective factor for mental healthiness (Taylor and Brown, 1988). We should note here that an associative analysis has also been useful in explicating the effects of psychopathology on these basic cognitive processes, providing explanations for changes in the psychological mechanisms underlying perceived control when people experience disorders like depression (see for example, Haselgrove and Hogarth, 2012). Later on in this paper, we too will utilize the associative framework in our discussion of the findings as we have done elsewhere (Msetfi et al., 2005).

### Perceived control and values

The traditional interpretation of the relation between perceived control and depression symptoms is based on the importance that is ascribed to “being in control.” In other words, feeling “in control” is psychologically valued to the extent that healthy people will such protect such feelings in the face of an uncontrollable situation (Alloy and Tabachnik, 1984). Conversely, the healthy bias is either absent (realism) or a negative bias is present either as a cause or a consequence of depression. Thus, control is of such psychological value that its absence is correlated with psychological disorder (Langer, 1975; Seligman, 1975). However, it is not clear whether the presence or absence of control would be psychologically ‘threatening’ if control were less valued.

Other work has explored these ideas using values associated with different cultural perspectives as an instance of between groups differences in values. One frequently made distinction is that of individualist vs. collectivist values (Hofstede, 1980; Triandis, 1996). Individualism, linked to Western culture, is associated with viewing the self as independent, with clear value placed on having personal goals and personal control. Collectivism tends to be related to Eastern culture in which the self is viewed as interdependent; value is placed on community and group goals. This simplistic description suggests that cultural distinctions are very clear cut when they are not (Cooper and Denner, 1998; Triandis and Gelfand, 1998; Green et al., 2005). It does suggest, however, that the importance and value placed on personal control is not universal (Markus and Kitayama, 1991) with corresponding implications for mental health. Moreover, interpretations of findings, in terms of optimistic illusory control and depressive realism, may not generalize to people with a different set of values.

Experimental work has shown that culture does influence learning about contingencies. For example, judgments made by Chinese and American students tested at an American university were different, though the nature of the difference was task dependent (Ji et al., 2000). When the task concerned passively experienced predictive contingencies, Chinese students' judgments tended to be higher than American students' judgments. No differences were observed in a subsequent task involving control contingencies. These findings are consistent with the idea that values affect contingency learning. However, that study was not designed to explore the relation between perceived control and mood, so further study is certainly required.

Although cross-cultural comparisons have been useful in identifying differences in contingency judgments, and other cognitive processes, this methodology may not be the best tool to address the questions we are interested in. There is considerable variability in the extent to which particular values are endorsed within specific settings. For example, there are differences between Scandinavian, British and US individualism (Triandis and Gelfand, 1998) and people can be both individualistic and collectivistic at the same time (Green et al., 2005). Moreover, globalization, demographic change and immigration create considerable diversity within settings and ethnic groups that might have previously been considered to be culturally uniform (Cooper and Denner, 1998). This variability points toward the importance of studying the effects of values on perceived control within cultural settings.

Therefore, we proposed to use the constructs of individualism and collectivism in order to identify the values held by our participants that are relevant to perceived control, and conduct our tests within a European setting. First, we planned to test whether such values have an effect on contingency learning in a similar manner to cross cultural differences (Ji et al., 2000). However, we also planned to test the effects of values on illusory control effects specifically because the absence of this effect is linked to depression (Alloy and Abramson, 1979). Thus, the first hypothesis was that (1) self-reported individualistic and collectivistic values will influence illusory control effects on contingency judgments. People with highly individualist values will value control to the extent that they evidence a stronger illusory control effect (greater difference between low and high density conditions) than those endorsing low individualist or collectivist values who value personal control to a lesser degree.

The second (2) hypothesis was that the relationship between contingency judgments and measures of mood state would depend on individualist and collectivist values. This hypothesis can be tested in two ways. The first is a moderation hypothesis (2a) in that “values” constitute variables which affect the direction or strength of the relation (Baron and Kenny, 1986) between perceived control and symptoms. In other words, those who experience a high level of control and value control, and those who experience low levels of control but don't value it, will experience lower levels of symptoms. However, where there is dissonance between experienced control and values there will be higher levels of symptoms. However, it is also possible that values mediate the relation between perception of control and symptoms. As Baron and Kenny argue, mediator variables explain how external events take on psychological significance (p. 1174) and provide an explanation for the relation. Thus we also plan to test the hypothesis (2b) that the relation between perceived control and symptoms is mediated by endorsed values.

These hypotheses were tested using a standard contingency learning task presented online in order to achieve the large sample size and adequate power. Variations in mood were measured in several different ways. Depressed mood has been repeatedly linked to low levels of perceived control (Seligman, 1975; Alloy and Abramson, 1979). We also measured the symptoms of bi-polar disorder, which is a mood disorder characterized by episodes of depression and elation. Bipolar disorder is pertinent here as it has been linked to high levels of perceived control, goal directed over-activity and optimism about the likelihood of producing outcomes (Langer, 1975). Consistent with this, induced elated mood has been shown to produce high levels of perceived control in depressed participants (Alloy et al., 1981).

## Experiment 1

### Method

#### Participants

Following approval from the Faculty Ethics committee (Approval number: EHSREC10-107), participants were recruited through the email systems of the University of Limerick in the Republic of Ireland. The survey remained open until no responses had been received for 2 weeks. Whilst 257 participants entered the online survey, 127 (49 males and 78 females) completed all questionnaires and the contingency learning task and were included in the data analyses. Of the final sample, 106 participants self-reported as white or white Irish, the remaining (*n* = 21) were of Asian or mixed origins. All participants declared their place of birth to be a Western country (e.g., Ireland, UK, USA, Australia, Europe), except one, who reported Japan as their place of birth.

Participants were on average 22 years old (*M* = 22.39, *SE* = 0.63) with 15 years of full time education (*M* = 15.03, *SE* = 0.20). Scores on the Beck Depression Inventory (BDI: Beck et al., 1961) and the Mood Disorders Questionnaire (MDQ: Hirschfeld et al., 2000), were in the expected range (Range_BDI_ = 0–50, *M*_BDI_ = 9.55, *SE*_BDI_ = 0.82; Range_MDQ_ = 0–13, *M*_MDQ_ = 5.62, *SE*_MDQ_ = 0.31). Average horizontal and vertical individualism scores were 10.52 (*SE* = 0.41) and 16.43 (*SE* = 0.48), respectively, whereas horizontal and vertical collectivism scores were 10.74 (*SE* = 0.37) and 11.37 (*SE* = 0.45), respectively.

#### Design

Two quasi-experimental designs were used in this study. The first involved two between groups independent variables: collectivism (low, high) and individualism (low, high). Two repeated measures variables were also manipulated, outcome density (OD: low 0.25, high 0.75 see Table 1), and cue (action, context). The latter variable refers to whether participants were rating their own control over the outcome via the action or the control exerted by the context. The dependent variable was ratings of control. This yielded a mixed fully factorial 2 × 2 × (2 × 2) design. The power analysis showed that for a medium effect, *f* = 0.25 and alpha = 0.05, this design requires *N* = 116 to achieve a power of 0.80. Order of condition presentation was counterbalanced.

The second part of this study involved ratings of control in the high OD condition in which healthy people tend to over-estimate their control and people with depression do not. In order to test Hypothesis 2a, we categorized participants as making low or high control judgments (see Data Analysis Section). Along with the other between groups variables, individualism (low, high) and collectivism (low, high), this yielded a fully factorial 2 × 2 × 2 design. The power analysis showed that for a medium effect, *f* = 0.25 and alpha = 0.05, this design requires *N* = 128 to achieve a power of 0.80. The aim of this was to assess whether the relationship between pre-existing tendencies toward low or high judgments of control and measures of mood state (BDI, MDQ) is affected by the endorsement of specific values. The final aspect of this design was correlational. Again, this specifically explored judgments of control in the high outcome density zero contingency condition only. The aim was to assess the extent to which any relationships between judgments of control and measures of mood related symptoms were mediated by endorsement of values.

#### Measures

##### Contingency task

The experimental task was programmed using java based code hosted at JudgementExperiment.com and presented using any standard web browser.

##### Beck depression inventory (Beck et al., 1961)

The BDI is a self-report measure of depressed mood used with both clinical and student populations (Bumberry et al., 1978). Each of the 21-items includes statements relevant to depression (e.g., I do not feel like a failure, I feel I am a complete failure as a person) scored up to a value of 3. Total scores can range from 0 to 63 with higher scores indicating higher levels of depression.

##### The mood disorder questionnaire (Hirschfeld et al., 2000)

The MDQ is a self-report questionnaire designed to be used as a screening tool for bipolar disorder. There are 13 items related to symptoms of mania that can be answered yes or no, with a yes answer yielding a score of 1. Subsequent questions then enquire about the frequency and significance of the consequences of the items mentioned previously. Only the initial 13 items were scored and summed to produce a total score as in other studies that used a general population sample (Dodd et al., 2010).

##### The individualism and collectivism scale (IC: Triandis and Gelfand, 1998)

The original scale included 32 self-report items on the endorsement of individualist and collectivist values. Here, we used the 16 items that have previously been found to have the highest factor loadings. There are four subscales—Horizontal Individualism (HI), Vertical Individualism (VI), Horizontal Collectivism (HC), and Vertical Collectivism (VC), where horizontal dimensions emphasize equality and vertical subscales emphasize hierarchy. People with a more prominent HI pattern value self-reliance and uniqueness, whereas those for whom VI is more prominent are more likely to place value on distinctiveness and high status. People who endorse more HC items value interdependence, sociability, and shared aims. Vertical collectivists strongly emphasize the importance of in-group integrity even at the cost of their own interests. Ratings were made on a 7-point scale ranging from 1 indicating “strongly disagree” to 7 indicating “strongly agree,” with a possible range of 4–28 for each subscale. Reliability was found to be at acceptable to good levels, with Cronbach's alpha calculated as α = 0.77, 0.76, 0.75, and 0.82 for the HI, VI, HC, and VC scales, respectively.

#### Procedure

Participants clicked on a link in an email and were taken to the online questionnaires and experimental task. After giving informed consent, participants were asked to provide demographic information, including age, gender, education, ethnic origin, nationality and country of birth. Then the BDI, MDQ, and IC scales were completed, in the same order for all participants, followed by the contingency task. The instructions informed participants that we are working on developing an online game and that we are interested in trying to understand levels of control in the way that people play all the different aspects of the game. In this particular test, participants would enter the “game testing room” and would be asked to try to make a ping sound occur on the computer inside the room (see Appendix 1 in Supplementary Material) by pressing the spacebar on the keyboard when signaled to do so by the appearance of a blue triangle on the computer screen. Each condition included 40 trials composed of a 1s response time window during which the blue triangle appeared on the screen. This was followed by the ping sound lasting for 0.5s at a probability of 0.75 following a response and a probability of 0.75 following no response in the high OD condition. In the low OD condition both probabilities were 0.25. Each trial was separated from the next by a 2s inter-trial interval during which the game testing room was visible on the computer screen. Following each condition, participants would be asked to make a judgment of their own control over the ping sound using the space bar, and then on the next screen a judgments about the control that the context (room, computer console etc.) exerted over the ping. Both judgments were made on a slider, which produced ratings that varied from 100 (total control) through 0 (no control) to −100 (totally prevent). On initial view, the “start position” of the slider was not shown; it only appeared once the participant had clicked into the slider to indicate the position on the scale of the final judgment. This was to ensure that the initial positioning of the slider did not bias participants' ratings. The full text of the instructions and the judgment screens is shown in Appendix 2 of Supplementary Material. Following completion of both conditions, participants were redirected to a thank you screen containing debriefing information and links to support information.

#### Data analysis

Three sets of analyses were conducted. The first analysis was designed to test whether the size of illusory control effects, the difference in judgments between the two OD conditions, varied as a function of individualistic and collectivistic values. We planned to categorize participants as low or high scorers on the IC scale. However, scores on the individualism and collectivism subscales were significantly positively correlated, *r*_(125)_ = 0.29, *p* = 0.001 (see Table A3.1 in Supplementary Material). This is not unusual and most agree that individualism and collectivism are not mutually exclusive (Green et al., 2005). Only scores on the vertical individualism (VI) and horizontal collectivism (HC) sub-scales were uncorrelated, *r*_(125)_ = 0.02, *p* = 0.82. We therefore used median splits on these subscales (VI median = 16, HC median = 10) in order to categorize participants, using the assumption that a low score represented lack of endorsement of the value rather than disagreement. This resulted in groups labeled: low individualist—low collectivist (*n* = 28); low individualist—high collectivist (*n* = 34), high individualist—low collectivist (*n* = 30); and high individualist—high collectivist group (*n* = 35). These group classifications were used as the between subjects variables.

The second analysis (Hypothesis 2a) was to test whether values moderated the relation between low and high control judgments and mood symptoms. To this end, we performed a median split on the judgment of control data in the high OD condition (median = 3) yielding a low control group (*n* = 65, *M* = −12.74, *SE* = 3.36) and a high control group (*n* = 62, *M* = 43.16, *SE* = 3.13). The final analysis was used to test Hypothesis 2b on the mediating role of values. The second set of analyses was intended to assess whether culture mediated any relation between control judgments and measures of psychopathology (mood). We used a non-parametric bootstrapping procedure (Preacher and Hayes, 2008), which estimates the indirect effects of multiple mediator variables, and in this case was based on 1000 bootstrap resamples with 95% bias corrected confidence intervals. These analyses were used to test the extent to which culture mediated the relation between contingency judgments of measures of psychopathology.

### Results and discussion

Participants completed a contingency judgment task in which they assessed their own control (action) and that of the context over the occurrence of an auditory outcome. These data were analyzed in relation to the two hypotheses and an alpha level of 0.05 was used throughout unless stated otherwise.

#### Hypothesis 1: illusory control effects are influenced by values

**Figure 1:**
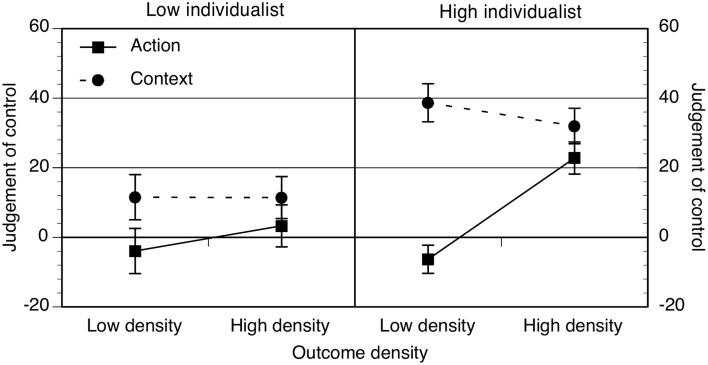
**Action and context ratings as a function of low and high outcome density condition and low or high individualist groups**. Error bars correspond to the standard errors of the mean.

Mean ratings of perceived control are shown in Figure 1 for the low and high individualist groups only (collectivism had no effect on judgments).

Key effects (see ANOVA Table 2) are that high outcome density ratings were higher than low outcome density ratings and that action ratings were significantly lower than context ratings. Both main effects were qualified by a significant cue × density interaction and a cue × density × individualism interaction. The simple interactions showed that high individualists' ratings strongly distinguished between action and context, *F*_(1, 61)_ = 24.10, *p* < 0.001, η^2^ = 0.28, and this effect was weaker for low individualists, *F*_(1, 58)_ = 4.59, *p* = 0.04, η^2^ = 0.07. For high individualists, there was a significant outcome density × cue interaction, *F*_(1, 61)_ = 15.23, *p* < 0.001, η^2^ = 0.20, but not for low individualists, *F* < 1. This showed that, for high individualists, their discrimination between action and context was present only in low outcome density conditions, and their action ratings increased significantly with higher levels of outcome density, high OD > low OD: *p* < 0.001. This was not the case for low individualists, *p* = 0.28. However, it is also important to note the presence of a cue × density × individualism × gender interaction. Further analysis showed that, in contrast to all other groups, low individualist male participants action ratings were un-affected by outcome density, *p* = 0.86, η^2^ = 0. These findings are generally consistent with our hypothesis that those who valued control would show larger illusory control effects than those who value it less.

**Table 2:** **Analysis of variance for judgments of control in Experiment 1 (Hypothesis 1)**.

**Source**	***F***	**η^2^**	***P***
**WITHIN SUBJECTS EFFECTS**			
**Cue**	**23.03**	**0.162**	<**0.001**
Cue × VI	3.56	0.029	0.062
Cue × HC	0.96	0.008	0.329
Cue × Gender	3.20	0.026	0.076
Cue × VI × HC	0.06	< 0.001	0.815
Cue × VI × Gender	1.64	0.014	0.203
Cue × HC × Gender	0.61	0.005	0.438
Cue × VI × HC × Gender	1.53	0.013	0.219
Error (Cue)	(1761.96)		
**Density**	**8.60**	**0.067**	**0.004**
Density × VI	2.30	0.019	0.132
Density × HC	1.25	0.01	0.265
Density × Gender	0.22	0.002	0.642
Density × VI × HC	1.05	0.009	0.307
Density × VI × Gender	0.98	0.008	0.325
Density × HC × Gender	0.11	0.001	0.742
Density × VI × HC × Gender	0.01	< 0.001	0.93
Error (density)	(682.18)		
**Cue** **×** **Density**	**10.69**	**0.082**	**0.001**
**Cue** **×** **Density** × **VI**	**4.66**	**0.038**	**0.033**
Cue × Density × HC	1.01	0.008	0.317
Cue × Density × Gender	0.32	0.003	0.576
Cue × Density × VI × HC	3.12	0.026	0.08
**Cue** **×** **Density** **×** **VI** **×** **Gender**	**5.11**	**0.041**	**0.026**
Cue × Density × HC × Gender	1.32	0.011	0.253
Cue × Density × VI × HC × Gender	0.50	0.004	0.482
Error (Cue × Density)	(1166.67)		
**BETWEEN SUBJECTS EFFECTS**			
**VI**	**11.64**	**0.089**	**0.001**
HC	0.038	< 0.001	0.845
Gender	2.972	0.024	0.087
VI × HC	0.015	< 0.001	0.903
VI × Gender	1.946	0.016	0.166
HC × Gender	0.039	< 0.001	0.843
VI × HC × Gender	0.108	0.001	0.743
Error	(2425.251)		

*This was 2 × 2 × 2 × (2 × 2) mixed factorial analysis of variance, with individualism (VI: low, high) and collectivism (HC: low, high) entered as between subjects factors, and Cue (action, context) and outcome density (low, high) entered as repeated measures factors. Gender was also included in the analysis as a between subjects factor. Mean squared error values are shown in parentheses. Bold indicates significant effects. Degrees of freedom = 1, 119*.

#### Hypothesis 2a: values moderate the relation between perceived control and symptoms

**Figure 2:**
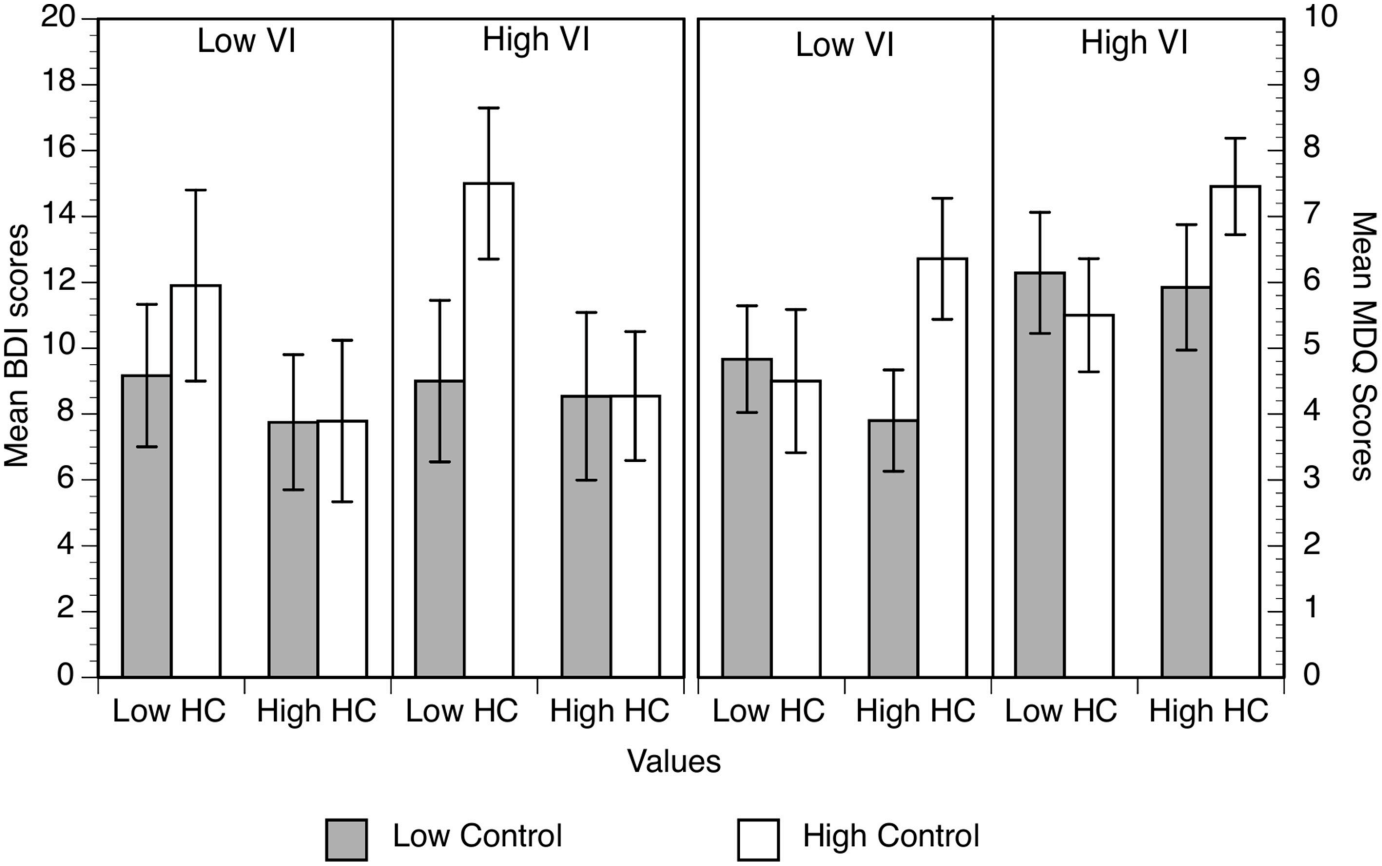
**Mean BDI (left) and MDQ scores (right) as a function of perceived control, individualist and collectivist values**. Error bars represent the standard error of the mean.

BDI and MDQ scores are shown in Figure 2 as a function of high and low perceived control and high and low values endorsement. Figure 2 suggests, contrary to our expectations, that high control participants experience higher levels of mood disturbance symptoms. However, the ANOVA showed there were no reliable effects on BDI scores (Table 3) but that high individualists produced higher MDQ scores than low individualists.

**Table 3:** **Analysis of variance on depression and bipolar scores in Experiment 1 (Hypothesis 2a)**.

**Source**	**Dependent variable:**			**Dependent variable:**		
	**BDI**			**MDQ**		
	***F***	**η^2^**	***p***	***F***	**η^2^**	***p***
Control	1.72	0.014	0.193	1.44	0.012	0.233
VI	0.45	0.004	0.505	**4.68**	0.038	**0.033**
HC	3.45	0.028	0.066	1.12	0.009	0.292
Control × VI	0.23	0.002	0.630	0.24	0.002	0.624
Control × HC	1.68	0.014	0.197	3.91	0.032	0.050
VI × HC	0.04	< 0.001	0.837	0.10	0.001	0.747
Control × VI × HC	0.24	0.002	0.624	0.06	0.001	0.807
Error	(−84.19)			(−11.81)		

*These were 2 × 2 × 2 ANOVAs with individualist (VI: Low High), collectivist (HC: Low, High) and Control (Low, High) as between subjects factors. Mean square error values are shown in parentheses. Bold indicates significant effects. Degrees of freedom = 1, 119*.

#### Hypothesis 2b: values mediate the relation between perceived control and symptoms

**Figure 3:**
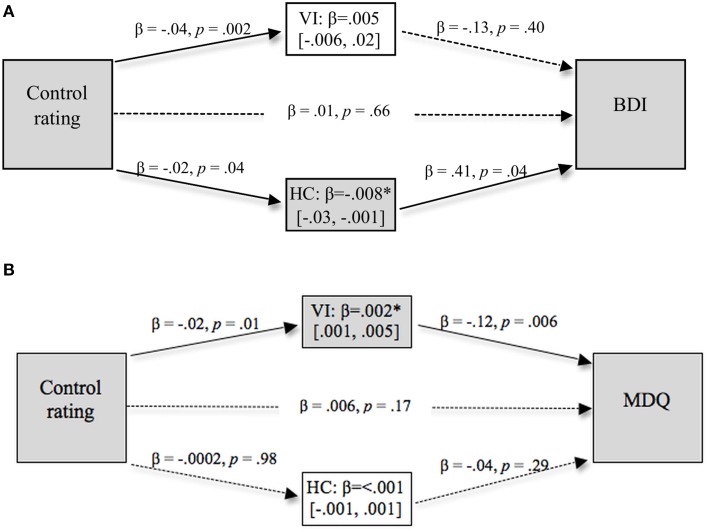
**Values as a mediator of the relationship between judgment of control and measures of (A) depression and (B) bi-polar symptoms**. Significant indirect pathways are emphasized with gray shading. Ninety-five percentage confidence limits around the indirect effects are shown in brackets.

The mediation models with BDI scores and MDQ scores as the outcome variables are shown in Figure 3, with significant direct pathways signified by solid lines and non-significant pathways signified by broken lines.

This analysis showed that the direct relationship between control rating and BDI scores was not reliable, β = 0.01, *p* = 0.66, however, the indirect pathway through HC scores was reliable at the 95% confidence level, β = −0.008 [−0.03, −0.001]. Thus while lower levels of perceived control were associated with higher levels of depression, this was completely mediated through higher endorsement of collectivist values.

The direct relationship between control ratings and MDQ scores was not reliable, β = 0.02, *p* = 0.16. However, the indirect pathway from control through VI to MDQ scores was reliable at the 95% confidence level, β = 0.005 [0.001, 0.01]. This was a complete mediation effect, and shows that higher levels of judged control predict higher MDQ scores and bipolar symptomatology, through lower endorsement of VI values.

Overall, Experiment 1 provided some evidence for the effects of values on contingency learning and symptoms. Individualism affected the size of illusory control effects and the extent to which people distinguished between their own control and that of the context. The analysis of depression symptoms was consistent across moderator and mediator analyses, no direct relation was evident between control and BDI scores. However, consistent with that, the mediation analysis suggested that HC values completely mediate the pathway between perceived control and depression symptoms. In addition, both the moderation and mediation analysis suggested that people with more individualist values tended to report higher levels of bipolar symptoms. The mediation analysis showed that judged control predicted bipolar symptoms via the value ascribed to that control. These findings provide support for the hypothesis that values are part of the psychological mechanism through which varying levels of control lead to mood disturbance.

The final sample of *N* = 127 was sufficient to detect medium effect sizes in the basic design with 80% power suggesting that the study was adequately powered. However, the sample lacked diversity and perhaps sufficient variability in values as only 16.5% of the final sample self-categorized as other than white or white Irish. Therefore, in the next experiment we aimed to replicate and extend the findings with a larger but more diverse sample.

In addition, in Experiment 1, we collected data on depression levels using the BDI. Whilst this is reliable measure of depression, BDI scores are also strongly correlated with levels of stress and anxiety (Lovibond and Lovibond, 1995), which can be a confound. Therefore, in Experiment 2, we chose to measure depression using an alternative self-report scale that distinguishes between depression, anxiety and stress.

## Experiment 2

In this experiment, we used the same procedure as the previous experiment to collect data from a larger, more diverse sample as well as using a measure of depression which distinguished between depression and correlates such as anxiety and stress.

### Method

Only details that are different to Experiment 1 are provided.

#### Participants

Following ethical approval from the School of Psychology Ethics Committee at the University of Hertfordshire, participants were recruited through the email systems of that UK University. Six hundred and sixty three participants entered the survey and 324 (122 males and 202 females) completed all questionnaires and the contingency learning task. Of the final sample, 165 (51%) self-reported themselves to be white, the rest were of Asian, black or of mixed origins. One hundred and sixteen (36%) participants indicated that they did not identify with the dominant culture, and 44 reported their place of birth to be a country often associated with collectivist culture (China, India, Pakistan, Iran, Malaysia etc.), the remainder were born in the UK and other Western countries.

Participants were on average 25 years old (*M* = 25.36, *SE* = 0.50) with 16 years of full time education (*M* = 15.84, *SE* = 0.13). Average scores on the Depression Anxiety Stress scales were 4.65, 3.33, and 5.46, respectively (*SE*s = 0.25, 0.20, 0.25) with scores within the expected range. Scores on the MDQ ranged from 0 to 13 (*M*_MDQ_ = 4.8, *SE* = 0.19). Average horizontal and vertical individualism scores were 9.67 (*SE* = 0.23) and 15.6 (*SE* = 0.30), respectively, whereas horizontal and vertical collectivism scores were 10.79 (*SE* = 0.23) and 11.27 (*SE* = 0.26), respectively.

#### Design

Participants were categorized on the VI and HC subscales (see detailed correlational analysis in Table A3.2 in Supplementary Material) and judgment of control ratings on the same basis as the previous experiment. The median value for control judgments was 13.5. Analyses were carried out as in Experiment 1.

#### Measures

##### Depression anxiety stress scales (DASS: Lovibond and Lovibond, 1995)

The DASS is a 42-item self-report questionnaire that yields three subscales measuring the symptoms of depression, anxiety and stress. Each item is rated on a scale of 0–3 indicating the extent to which that item had applied to the participant over the past week. There are 14 items for each emotional state with a maximum possible score of 42.

### Results and discussion

#### Hypothesis 1: illusory control effects are influenced by values

**Figure 4:**
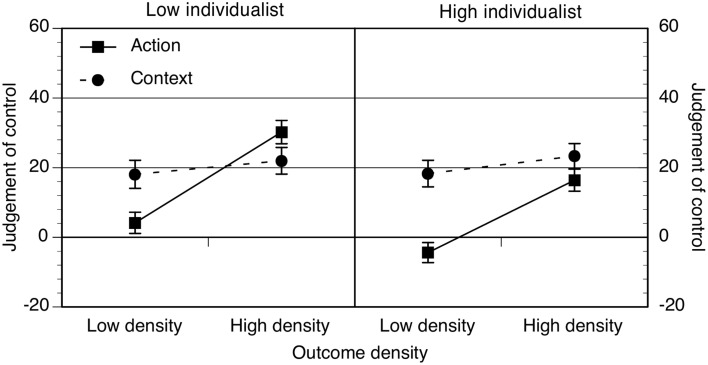
**Action and context ratings as a function of low and high outcome density condition and low or high individualism groups**. Error bars correspond to the standard errors of the mean.

Participants' judgments of control were analyzed as previously. Gender did not interact with any of the other variables and was excluded from the analysis. The data are shown in Figure 4 and the ANOVA in Table 4.

**Table 4:** **Analysis of variance for judgments of control in Experiment 2 (Hypothesis 1)**.

**Source**	***F***	**η^2^**	***p***
**WITHIN SUBJECTS EFFECTS**			
**Cue**	**10.56**	**0.032**	**0.001**
**Cue** × **VI**	**4.85**	**0.015**	**0.028**
Cue × HC	1.43	0.004	0.233
Cue × VI × HC	0.22	0.001	0.640
Error(Cue)	(2338.55)		
**Density**	**47.40**	**0.129**	<**0.001**
Density × VI	0.26	0.001	0.608
Density × HC	0.15	< 0.001	0.701
Density × VI × HC	1.11	0.003	0.293
Error (density)	(1299.63)		
**Cue** × **Density**	**24.93**	**0.072**	<**0.001**
Cue × Density × VI	0.70	0.002	0.403
Cue × Density × HC	0.27	0.001	0.607
Cue × Density × VI × HC	0.02	< 0.001	0.876
Error (Cue × Density)	(1146.14)		
**BETWEEN SUBJECTS EFFECTS**			
VI	2.92	0.009	0.088
HC	0.03	< 0.001	0.855
VI × HC	4.82	0.015	0.029
Error	(2935.21)		

*This was 2 × 2 × (2 × 2) mixed factorial analysis of variance, with individualism (VI: low, high) and collectivism (HC: low, high) entered as between subjects factors, and Cue (action, context) and outcome density (low, high) entered as repeated measures factors. Mean squared error values are shown in parentheses. Bold indicates significant effects. Degrees of freedom = 1, 320*.

Participants made higher ratings with higher levels of outcome density, although the size of this effect depended on whether the action or context was rated. Further analysis showed that action judgments were sensitive to outcome density, *p* < 0.001, η^2^ = 0.18, but context ratings were not, *p* = 0.12, η^2^ = 0.008. Action ratings tended to be lower than context ratings but the size of this effect depended on levels of individualism. Low individualist participants attributed a similar degree of control to the action and the context, *p* = 0.47, η^2^ = 0.002. However, high individualist participants' context ratings were significantly higher than their action ratings, *p* < 0.001, η^2^ = 0.05.

Finally, there was a significant interaction between individualism and collectivism. Overall, high individualists tended to produce lower ratings (*M* = 10.34, *SE* = 3.15) than low individualists (*M* = 22.22, *SE* = 3.33) but this was only the case for high individualists who were also low collectivists, *p* = 0.01, η^2^ = 0.02, and not those who were also high collectivists, *p* = 0.71.

#### Hypothesis 2a: values moderate the relation between perceived control and symptoms

**Figure 5:**
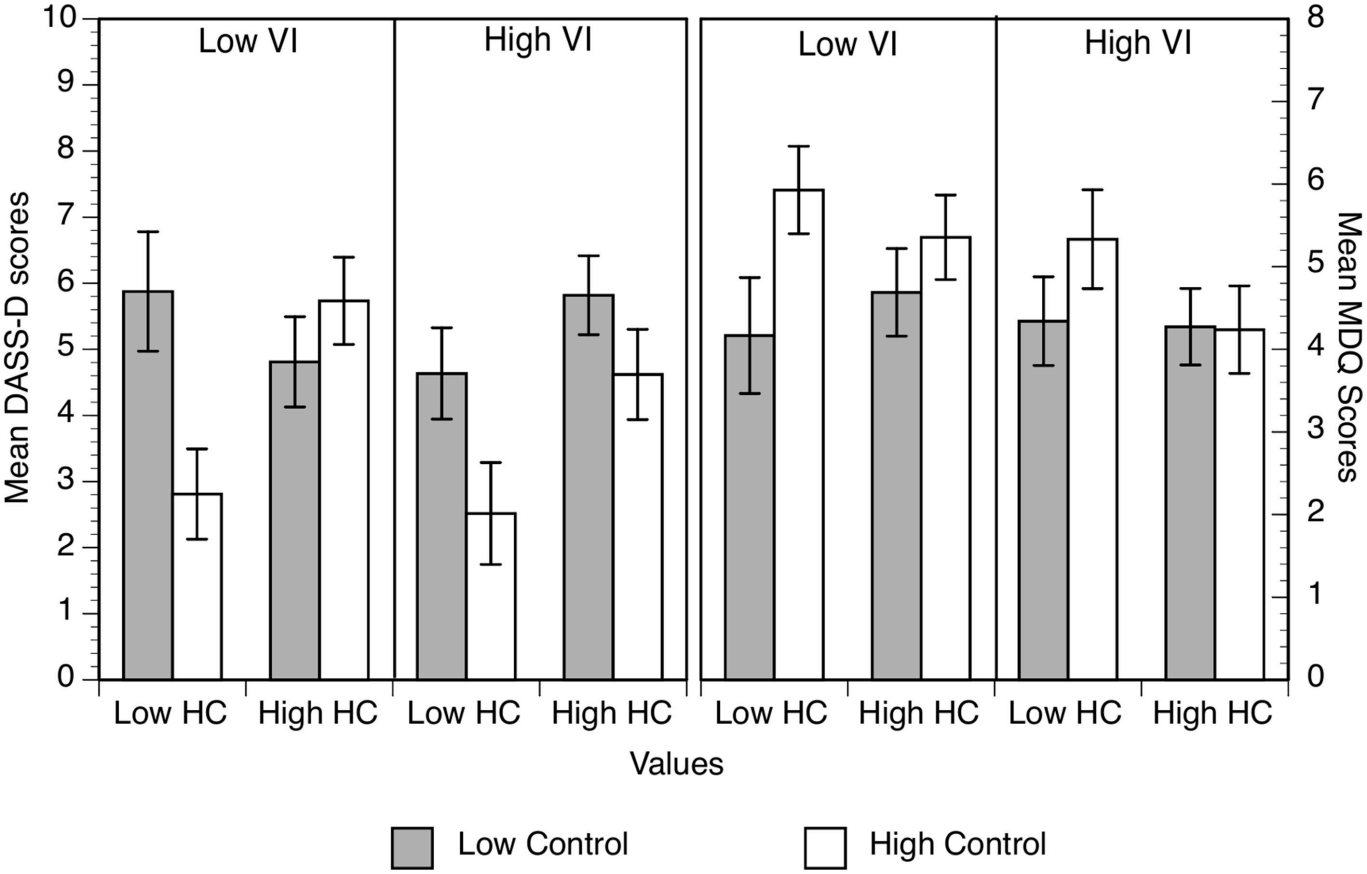
**Mean DASS-D (left) and MDQ scores (right) as a function of perceived control, individualist (VI) and collectivist (HC) values**. Error bars represent the standard error of the mean.

Depression (DASS-D) and MDQ scores are shown in Figure 5 with the ANOVA in Table 5.

**Table 5:** **Analysis of variance on depression and bipolar scores in Experiment 2 (Hypothesis 2a)**.

	**Dependent variable:**			**Dependent variable:**		
	**DASS-D**			**MDQ**		
**Source**	***F***	**η^2^**	***p***	***F***	**η^2^**	***p***
Control	**7.31**	**0.023**	**0.007**	**4.66**	**0.015**	**0.032**
VI	0.66	0.002	0.417	1.56	0.005	0.213
HC	**6.49**	**0.020**	**0.011**	0.60	0.002	0.440
Control × VI	0.34	0.001	0.561	0.88	0.003	0.349
Control × HC	**5.91**	**0.018**	**0.016**	1.83	0.006	0.177
VI × HC	0.50	0.002	0.480	0.51	0.002	0.478
Control × VI × HC	2.31	0.007	0.130	< 0.01	< 0.001	0.964
Error	(19.60)			(11.81)		

*These were 2 × 2 × 2 ANOVAs with individualist (VI: Low High), collectivist (HC: Low, High) and Control (Low, High) as between subjects factors. Mean square error values are shown in parentheses. Bold indicates significant effects. Degrees of freedom = 1, 316*.

There were main effects of perceived control and collectivism on depression scores, that were qualified by a control × collectivism interaction. Subsequent simple effects analyses showed that for low collectivists only, low perceived control participants showed significantly higher levels of depression symptoms than participants who judged that they had high levels of perceived control, *F*_(1, 316)_ = 11.41, *p* = 0.001, η^2^ = 0.04. For high collectivists, perceived control had no effect on symptoms, *F* < 1, *p* = 0.8. The only variable that had a reliable effect on MDQ scores was perceived control. Participants reporting higher perceived control also reported higher symptoms levels than low perceived control groups.

#### Hypothesis 2b: values mediate the relation between perceived control and symptoms

**Figure 6:**
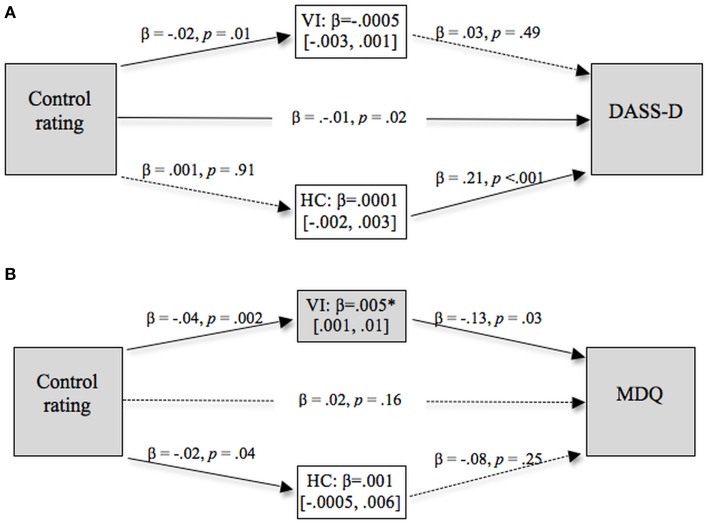
**Values as a mediator of the relationship between judgment of control and measures of (A) depression and (B) bi-polar symptoms, controlling for anxiety**. Significant direct pathways are indicated by solid (vs. broken) lines. Significant indirect pathways are emphasized with gray shading. Ninety-five percentage confidence limits around the indirect effects are shown in brackets.

Two mediator analyses were carried out and the results of these analyses are shown in Figure 6.

Judgments of control were negatively related to DASS depression scores (a), such that high levels of perceived control were related to low levels of depression and vice versa, β = −0.01, *p* = 0.02, a finding consistent with previous research on depressive realism. However, neither of the indirect effects were reliable, VI: β = 0.02 [−0.003, 0.001], HC: β = −0.0001 [−0.003, 0.001]. In contrast to Experiment 1, HC scores did not mediate the relation between judged control and depression.

Judgments of control did not show a direct relation with MDQ scores (b), β = 0.02, *p* = 0.16. However, there was a completely mediated relation between the two variables through VI scores, VI: β = 0.005 [0.001, 0.01], an effect significant at the 95% confidence level. Note that both sets of mediation analyses were carried out both with and without anxiety included as a statistical control and this did not change the pattern of results.

In summary, the findings of Experiment 2 showed that values influenced contingency judgments. Like Experiment 1, those who endorsed high individualist values showed greater discrimination between action and context ratings than people who did not endorse individualist values and this effect was dependent on levels of outcome density. In addition, values intervened in the relationship between perceived control and mood disturbance. Those who did not endorse collectivist values and perceived their lack of control reported higher levels of depression symptoms, this was a moderator effect rather than a mediated effect. Similar to Experiment 1, evidence showed that individualist values mediated and did not moderate the control—bipolar relation. Taken together, these findings suggest that values directly influence contingency learning and indirectly affect the relation to symptoms.

However, the two different samples did not produce identical results. While the mediating effect of individualist values in relation to bipolar symptoms was consistent, the moderating effect was not. Also the intervening effect of collectivist values was not consistent across the two experiments, whether mediating or moderating. These differences might be because the Irish sample was smaller and less diverse (*N* = 127) than the UK sample (*N* = 324), of which 36% said they did not identify with the dominant, arguably individualist, culture, possibly resulting in more consistency and similarity in values between Irish participants. In the sample in which most participants claimed an individualist background, individualist values had a direct impact on illusory control and symptoms. High levels were associated with stronger illusory control effects and higher levels of bipolar symptoms. In the sample in which more people were from a collectivist background, those who did not tend to endorse collectivist values and perceived higher levels of control self-reported the lowest symptoms of depression, although the mediated effect was not evident. Participants with highly collective values experienced higher levels of depression irrespective of control levels. This suggests that collectivist values consistent with lower control do not provide a simple protective shield from depression as we envisaged. In both samples, however, individualist values that were also consistent with lower control (i.e., individualist values not endorsed) amplified the positive predictive relation between control and bipolar symptoms.

## Experiment 3

Given the quasi-experimental and correlational nature of the designs used in Experiments 1 and 2, it is likely that the composition of the sample and variability in their values will influence the precise effects observed. In addition, given that these are essentially correlational findings, other unknown variables could be responsible for the effects (Oyserman and Lee, 2008).

Therefore, in order to extend these results, we decided to run an experimental study in order to manipulate experimentally both values and perceived control in a random sample of participants and measure the effects of this manipulation on immediate emotional state. First, participants would be primed with specific values (Hong et al., 2000; Gelfand et al., 2014) relevant to control, individualism and collectivism—in this case the value of winning vs. participation—and then be exposed to conditions which usually produce low or high levels of perceived control. Following this, momentarily experienced affect, both negative and positive, would be measured because values might exert their effect by enhancing or diminishing either type of affective state.

### Method

Only new information will be reported.

#### Participants

A total of 876 volunteers from the two universities in the UK and the Republic of Ireland entered the online survey, of which 272 participants completed all measures (106 males and 166 females). Of the final sample, 185 (68%) self-reported as white, and 73 (26.8%) reported that they did not identify with the dominant culture. On average, participants were 28 years old (*SE* = 0.73) and had received 16.07 years of education (*SE* = 0.15). Average scores on the BDI and the MDQ were 11.20 and 4.92 (*SE*_BDI_ = 0.52; *SE*_MDQ_ = 0.22), respectively.

#### Design

This experiment used a 2 × 2 between subjects design, where the first between subjects variable was primed values (winning, participating) and the second between subjects variable was outcome density (OD: low 0.25, high 0.75). Participants were also categorized as low or high individualist as in the previous experiments. Momentary levels of positive and negative affect following completion of the contingency task were the dependent variables. In addition, and in order to control for pre-existing levels of affect, validated cutoff scores on the BDI (Beck et al., 1961) were used to categorize participants as not depressed or mildly depressed (<9 not depressed *n* = 119, >8 mildly depressed *n* = 153) and entered into the analysis as a covariate (note that while the proportion of the sample showing signs of depression may seem high, this is consistent with previous work using the BDI, e.g., Bumberry et al., 1978, 40/56 student participants showed signs of depression; and the DASS, e.g., Bayram and Bilgel, 2008, 48% student participants showed signs of depression). As in previous experiments, judgments of control were also analyzed.

#### Measures

##### Positive and negative affect scales (PANAS: Watson et al., 1988)

The PANAS includes 20 words that describe aspects of positive and negative affect (10 positive, 10 negative). Participants are asked to indicate the extent to which they “feel this way right now, that is, in the present moment,” using a 5-point Likert scale which ranges from 1 (very slightly or not at all) to 5 (extremely). The possible range of scores is 10–50 for each subscale. This is one of the most widely used scales to measure affect and has received extensive validation (e.g., Watson et al., 1988; Crawford and Henry, 2004).

#### Procedure

The procedure was the same as the previous experiment with the addition of the priming instructions before the contingency task (see Appendix 4 in Supplementary Material for details) and the PANAS being completed afterwards.

### Results and discussion

Participants were primed to value winning or participating before taking part in the contingency task and then rated their levels of positive and negative affect. Pre-existing individualism was included in all analyses as a between groups variable.

#### Hypothesis 1: illusory control effects are influenced by values

**Figure 7:**
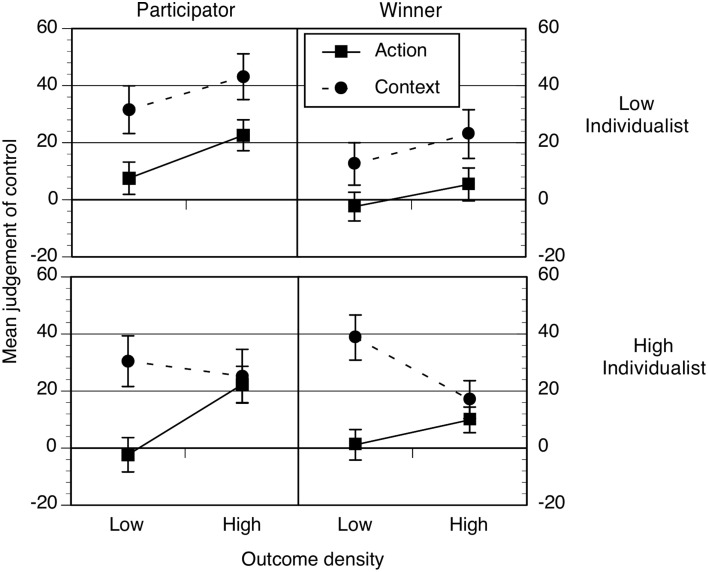
**Mean action and context ratings as a function of outcome density (OD), values prime (participate left, win right) and individualism (low top, high bottom)**. Error bars correspond to the standard errors of the mean.

Judgments are shown in Figure 7 and with the ANOVA in Table 6. This showed that context judgments were significantly higher than action judgments but this main effect was qualified by two further interactions: cue × outcome density and cue × outcome density × individualism. In addition, the values prime effect depended on levels of individualism. Follow up analyses showed that outcome density effects on action ratings were significant for high (*p* = 0.003, η^2^ = 0.03) and low individualists (*p* = 0.04, η^2^ = 0.02). Outcome density effects on context ratings were not reliable for either group, all *p*s > 0.1. Only high individualists showed the cue by density interaction, *p* = 0.002, and not low individualists, *p* = 0.97. Finally, the values prime had no influence on the ratings of high individualists (action and context), *p* = 0.65, but low individualist participators produced significantly higher ratings overall than winners, *F*_(1, 256)_ = 12.10, *p* = 0.001, *MSE* = 702.338, η^2^ = 0.05.

**Table 6:** **Analysis of variance on judgments of control in Experiment 3 (Hypothesis 1)**.

**Source**	***F***	**η^2^**	***p***
**WITHIN SUBJECTS EFFECTS**			
**Cue**	**30.241**	**0.106**	<**0.001**
**Cue** **×** **OD**	**4.549**	**0.017**	**0.034**
Cue × Prime	0.011	< 0.001	0.918
Cue × VI	0.013	< 0.001	0.910
Cue × Gender	3.873	0.015	0.050
Cue × OD × Prime	0.038	< 0.001	0.846
**Cue** **×** **OD** **×** **VI**	**4.317**	**0.017**	**0.039**
Cue × OD × Gender	1.432	0.006	0.233
Cue × Prime × VI	0.534	0.002	0.466
Cue × Prime × Gender	0.025	< 0.001	0.874
Cue × VI × Gender	2.509	0.010	0.114
Cue × OD × Prime × VI	0.056	< 0.001	0.813
Cue × OD × Prime × Gender	0.726	0.003	0.395
Cue × OD × VI × Gender	1.746	0.007	0.188
Cue × Prime × VI × Gender	0.063	< 0.001	0.802
Cue × OD × Prime × VI × Gender	0.312	0.001	0.577
Error (Cue)	(1552.21)		
**BETWEEN SUBJECTS EFFECTS**			
OD	3.52	0.014	0.062
**Prime**	**7.60**	**0.029**	**0.006**
VI	0.001	< 0.001	0.974
Gender	0.03	< 0.001	0.87
OD × Prime	2.24	0.009	0.136
OD × VI	2.00	0.008	0.159
**OD** × **Gender**	**5.40**	**0.021**	**0.021**
**Prime** × **VI**	**4.45**	**0.017**	**0.036**
Prime × Gender	1.43	0.006	0.233
VI × Gender	0.03	< 0.001	0.873
OD × Prime × VI	0.77	0.003	0.380
OD × Prime × Gender	0.32	0.001	0.571
OD × VI × Gender	0.02	< 0.001	0.891
Prime × VI × Gender	0.95	0.004	0.330
OD × Prime × VI × Gender	1.03	0.004	0.312
Error	(1404.68)		

*This was 2 × 2 × 2 × 2 × (2) mixed factorial analysis of variance, with prime (win, participate), individualism (VI: low, high) and outcome density (OD: low, high) and gender (male, female) entered as between subjects factors; cue (action, context) was entered as a repeated measures factors. Mean squared error values are shown in parentheses. Bold indicates significant effects. Degrees of freedom = 1, 256*.

#### Hypothesis 2a and 2b: values influence the relation between control and symptoms

**Figure 8:**
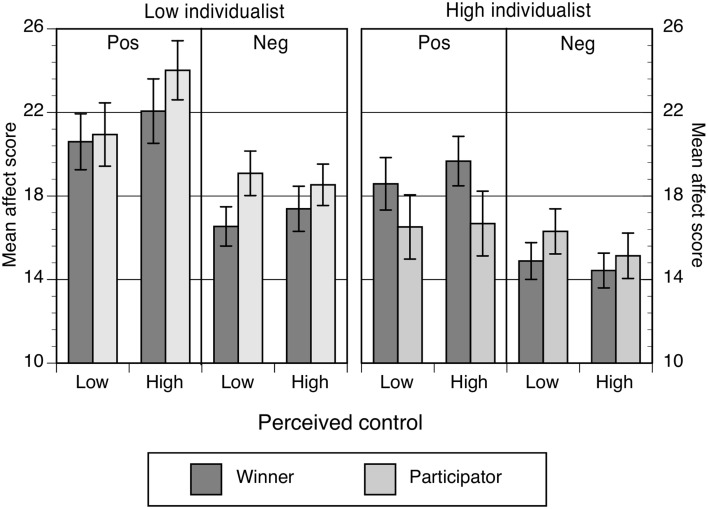
**Mean affect scores as a function of perceived control, values prime and levels of individualism**. Error bars correspond to the standard error of the mean. “Pos” refers to positive affect, whereas “Neg” refers to negative affect.

Ratings of positive and negative affect taken following the contingency task are shown in Figure 8, with the MANOVA controlling for depression levels in Table 7.

**Table 7:** **Analysis of variance on post task affect scores in Experiment 3 (Hypothesis 2)**.

**Source**	**Dependent variable: affect**	***F***	**η^2^**	***p***
**Depression group**	**Positive**	**11.58**	**0.042**	**0.001**
	**Negative**	**18.91**	**0.067**	<**0.001**
OD	Positive	2.09	0.008	0.150
	Negative	0.22	0.001	0.637
Prime	Positive	0.47	0.002	0.493
	**Negative**	**4.25**	**0.016**	**0.040**
**VI**	**Positive**	**15.58**	**0.056**	<**0.001**
	**Negative**	**14.10**	**0.051**	<**0.001**
OD ^*^ Prime	Positive	0.03	< 0.001	0.865
	Negative	0.55	0.002	0.457
OD ^*^ VI	Positive	0.67	0.003	0.413
	Negative	0.46	0.002	0.497
Prime ^*^ VI	Positive	3.36	0.013	0.068
	Negative	0.31	0.001	0.578
OD ^*^ Prime ^*^ VI	Positive	0.40	0.002	0.528
	Negative	0.06	< 0.001	0.809
Error	Positive	(65.92)		
	Negative	(32.42)		

*This was a multivariate analysis of variance with PANAS positive and negative scores entered as the two dependent variables; perceived control, values prime and individualism were entered between subjects independent variables. Depression group was included as a covariate. Mean square error values are shown in parentheses. Bold indicates significant effects. Degrees of freedom = 1, 263*.

Individualist values had a strong and consistent effect on both positive and negative affect, with low individualists producing higher affect scores on both dimensions, possibly indicating more willingness to acknowledge or report their emotions. However, the values prime independently increased levels of negative affect but had no effect on positive scores. Item level analysis showed that specific negative items were influenced by the prime. In comparison to winners, participators were significantly more upset (*p* = 0.05), scared (*p* = 0.05), hostile (*p* = 0.04), jittery (*p* = 0.017), and afraid (*p* = 0.014), but not distressed (*p* = 0.601), guilty (*p* = 0.324), irritable (*p* = 0.191), ashamed (*p* = 0.340), or nervous (*p* = 0.602). The prime effect did not depend on high or low levels of perceived control.

A supplementary mediation analysis was also carried out to check that the indirect pathway from high OD judgments of control through vertical individualism to MDQ scores observed in Experiments 1 and 2 was replicated here, and it was, β = 0.003 [0.000, 0.010]. Moreover, indirect pathways were also evident to negative affect scores, including jittery and afraid.

Taken together, the findings of Experiment 3 show that pre-existing values, but not primed values, systematically influence contingency learning, such that for high individualists judgments of the action and context were affected by outcome density. A similar pattern was observed in Experiments 1 and 2 but was much clearer in Experiment 3, possibly due to the between subjects nature of the design. In Experiment 3, however, the influence of values on the outcome density effect on action ratings was not reliable. This suggests that the overarching effect of pre-existing values on perceived control is not the simple one we predicted. Rather the effect of values is more complex and seems to be exerted through its influence on context ratings.

Importantly, in the context of Experiments 1 and 2, we showed experimentally that values influenced contingency learning and subsequent responses to that situation. Participants primed to value participation show higher levels of negative affect after the taking part in the contingency judgment task. This trend was focused on items related to anxiety as opposed to depressive affect, which is consistent with heightened anxiety when there is dissonance between task and values. In addition, and as might have been expected, both the values prime and levels of individualism influenced participants' judgments of control in a distinct manner. Individualism influenced the extent to which outcome density determined discrimination between action and context. Only participators', but not winners', judgments showed systematic evidence of increasing with higher levels of outcome density.

## General discussion

The findings reported here provide evidence for the influence of values on contingency learning and the relation between perceived control and symptoms of mood disturbance. Results suggest that the effects of pre-existing values on perceived control are transmitted through a consistent effect on perception of context rather than directly as a simple bias toward valuing control. In spite of this, we have also reported results here that suggest that there **is** a bias toward valuing control at play and that this bias influences mood disturbance and people's immediate responses to control tasks. These findings will be discussed in turn below, before acknowledging the limitations of this work and future directions.

### Values effects on learning and perceived control

The consistent effect that we noted in these experiments is that individualist values influence the relation between action and context ratings and outcome density. Across three experiments, high individualists discriminated strongly between their own control and context control when there were few outcomes (low outcome density). However, when many outcomes occurred, participants rated their own control as equal to that of the context. The same interaction was not strongly evident in low individualists. We argue that associative learning theories (e.g., Rescorla and Wagner, 1972; Pearce, 1987) provide a useful framework with which to explain these values effects because, according to this perspective, context plays a key role in contingency learning.

Associative learning theories can be categorized as involving configural or elemental processing, with different implications for how the context is processed. For example, the Rescorla–Wagner model (RW: Rescorla and Wagner, 1972) is an elemental learning model because it holds that every stimulus, including the context, becomes individually associated with the outcome when it occurs. These individual associations are strengthened when stimulus and outcome co-occur and are weakened when the stimulus occurs alone. As there is a finite amount of associative strength available for any given outcome, associative strength is accrued to the extent that it is available. The ‘associative competition’ implied by the model means that the action-outcome association will be weakened if the outcome gets associated with the context, for example when there are high levels of outcomes, and strengthened if it is not. This means that the relation between action and context associations is highly influenced by outcome density (Msetfi et al., 2005) in a manner similar to the patterns of action and context ratings we have observed in the high individualist participants. Thus, it could be argued that high individualists are displaying some evidence of elemental processing.

In contrast, configural associative learning models (e.g., Pearce, 1987) hold that learning involves acquiring holistic or configural representations of stimuli, including the context, and it is the configurations that enter into association with the outcome rather than the individual elements. It follows then that when stimuli are presented embedded in configurations (i.e., action in context) any associative strength accrued by the elements will generalize back to the configuration and vice versa based on their similarity. So the context and outcome become strongly associated to the extent that outcomes occur. This associative strength then generalizes back to the action-context-outcome association because of the similarity between the action-context configuration and the context alone. This means that the configural model does not predict an interaction between outcome density and action/context in the manner we have observed with high individualists. Rather the configural model predicts that the difference between the action and context associations will be constant over levels of outcome density. Thus our low individualist data have more in common with a configural learning process than an elemental one.

**Figure 9:**
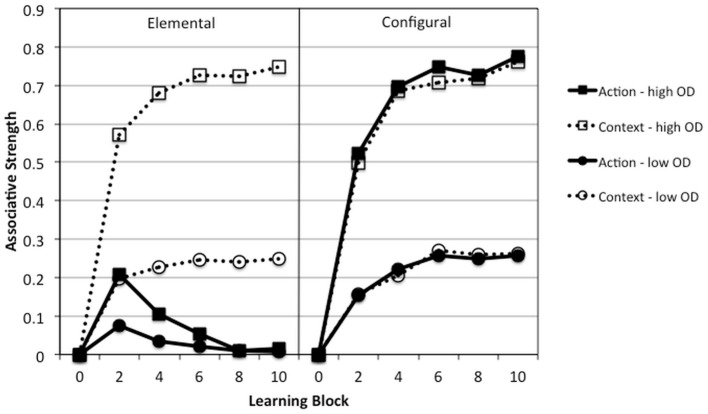
**Predictions of the Rescorla–Wagner (left panel) and Pearce models (right panel) for the acquisition over time and learning blocks of Action and Context associations (solid and broken lines) in low and high outcome density conditions (square and circle symbols)**. Acquisition functions were simulated over 10 learning blocks using a value of 0.2 for the α, β_1_, and β_0_ parameters.

Figure 9 shows these predictions, using Rescorla–Wagner (left) and Pearce model (right) simulations for which all learning rate parameters were set to be equal (α sets the salience of the action and the context, β sets the strength of the outcome). Note that adjustment of the learning rate parameters does not change the pattern of action-context discrimination predicted by the models. Thus data in this study are consistent with high individualists distinguishing between action and context in a manner most consistent with elemental processing, whereas low individualists seem to learn in a configural manner, in which actions are tied to the strength of the context. Although we also note that neither model predicts that this difference should decrease in high outcome density conditions, it fact the difference should increase. Overall, our findings are consistent with the idea that pre-existing individualist values, but not primed values, affect mode of processing, with high individualists showing evidence of elemental learning and low individualists' data being more suggestive of configural learning. In other words, learning processes are not absolute, either elemental or configural (Williams et al., 1994; Melchers et al., 2008; Byrom and Murphy, 2014).

This interpretation of the data is broadly in line with other evidence that cultural perspectives and values influence modes of information processing (e.g., Nisbett et al., 2001; Nisbett and Miyamoto, 2005). For example, when Chinese participants were exposed to Rorschach stimuli, they responded in a manner consistent with the perception of whole patterns, whereas American Chinese participants' responses focused on the components of the stimulus (Abel and Hsu, 1949). Thus, cross-cultural research has also suggested differences in perception between collectivist and individualist people, in terms of holistic processing of groups of stimuli vs. object focused processing of individual stimuli within groups (Nisbett et al., 2001; Nisbett and Miyamoto, 2005; Park and Huang, 2010). In other words, collectivist values are linked to configural processing and individualistic values to elemental processing.

It is important to note, however, that we only found evidence of an effect of individualist values on contingency learning, based on highly endorsing or being ambivalent about individualistic values. Stronger endorsement of collectivist values did not produce similar effects to weak endorsement of individualistic values. This is not entirely surprising given that the sample was recruited from a European, predominantly individualistic, culture. However, a contribution of this paper is to show that while values effects on information processing might have been thought of as a cross cultural phenomenon they are also evident in this relatively homogenous sample recruited from within European culture. Another novel contribution of this work is to demonstrate that associative learning theory can explain some of these effects. This is consistent with the idea that stimuli can be coded flexibly and that learning processes are not absolute, either elemental or configural (Williams et al., 1994; Melchers et al., 2008; Byrom and Murphy, 2014).

### Values influence the relation between control and symptoms

Values and perceived control were associated with signs of mood disturbance. These findings were not exactly as expected. For example, we anticipated that values would affect the relation between judgments of control and depressed mood because of the psychological value placed on control (Betancourt and Lopez, 1993) and because the consequences of perceived lack of control have consistently been related to depression (Seligman, 1975). We tested both (a) moderation and (b) mediation hypotheses in order to test (a) for a simple qualitative difference between value groups, and, given that intervening mediator variables can completely suppress evidence of a direct effect, to test (b) whether values indirectly influenced the control to symptom relation. The moderating effect of collectivist values on depression was only evident in the second, larger, relatively diverse, sample in which a depression measure was used that was intended to distinguish between anxiety and stress. Low levels of perceived control were associated with higher levels of depressed mood but only in those who did not endorse collectivist values. Evidence for the mediating effect of collectivist values on depression was only seen in the smaller less diverse sample in Experiment 1. Thus we can only conclude that any intervening effect of values on depression symptoms was very much influenced by sample composition.

However, one mediated pathway was strongly and consistently evident in all experiments and involved the euphoric symptoms of bipolar disorder as opposed to depression. Higher levels of perceived control (when people had none) were predictive of higher levels of such symptoms in a pathway through lower levels of vertical individualism. People reporting high perceived control and lower levels of vertical individualism also reported higher levels of mood disturbing symptoms related specifically to mania or elation. (Note that the measure used here, the MDQ, includes only items on the frequency of manic aspects of bi-polar symptoms over the long term and does not include items on low or depressive symptoms). This relationship was significant both when anxiety was controlled in the analysis and when it was not. These mediated pathways are interesting because they suggest that if control is perceived to be high, and low value is placed on it, this could lead to psychological disturbance.

As we were interested in the processes through which such effects might occur, it was also important to test for similar effects experimentally. Priming the value of participation in comparison to winning produced higher levels of negative affect, particularly anxiety, after the contingency task irrespective of the level of control perceived. While this negative influence of priming was not focused in high illusory control conditions only, we did find that control judgments in the same conditions indirectly predicted both bipolar symptoms and negative affect through the vertical individualism pathway. Taken together, these correlational and experimental findings provide strong support for the role of perceived control and cultural values in mood disturbance. It could also be argued that whereas primed values can exert a direct effect on the emotional response to a given task, it might be that values embedded in the task itself (e.g., Wigfield et al., 2004), rather than the degree of perceived control *per se*, create dissonance with primed values and thus anxiety. Therefore, an anxious emotional response to value-laden situations might be one pathway through which symptoms of mood disturbance eventually develop.

A link between perceived control and bipolar symptoms is not unanticipated. For example, Langer speculated that misattribution of causal effectiveness to the self, the so-called illusion of control could be an etiological factor in manic symptoms (Langer, 1975). The new finding here is that this link is completely mediated by cultural values, specifically vertical individualism. People who score highly in vertical individualism subscale endorse items related to the importance of winning and competitiveness, as well as social status comparisons related to being seen to do better than others and feeling quite anxious when the performance of others is evaluated as better than one's own (Triandis and Gelfand, 1998). People with low scores disagree with these values. Thus we found that a person who feels highly “in control” but disagrees with these competition and status values is more likely to be anxious and to experience euphoric mood disturbance. This would be consistent with an individual's perspective along the lines of, “I have high levels of control, and I don't care about success and what people think.” This evidence is consistent with the idea that “having control” is differentially desirable in terms of psychological wellbeing depending on particular cultural values.

Whilst this new finding does partially support our original hypotheses that feeling “in control” is not always a hallmark of being mentally healthy (see also Cheng et al., 2013), this study does not involve a clear contrast between individualist vs. collectivist values. This is because in these experiments, those who did not agree with [vertical] individualist values did not necessarily endorse collectivist values either. This may be partly because we chose to identify values in participants living within one cultural setting. This decision was based on the idea that hard and fast cultural distinctions between individualist and collectivist perspectives, while being very useful, are not absolute (Green et al., 2005). As many scholars have pointed out, every Western country or every Asian country will not have the same precise pattern of cultural values (e.g., Triandis and Gelfand, 1998). Our data supported that view. Two particular subscales of the individualism—collectivism questionnaire, but not all four, distinguished our participants. These were vertical individualism and horizontal collectivism, however, it was vertical individualism values that were endorsed most strongly by our samples in comparison to all other values. It was also vertical individualist values that were linked to different patterns of learning about control.

Taken together, these findings suggest that the psychological value ascribed to “having control” by theorists, researchers, and practitioners should not be considered to be a universal but as a function of the values that an individual does or does not hold, their own desired and perceived level of control, the specific situation in which they find themselves and the values relevant to that situation. In this study, all of these factors influenced mood and affective disturbance to some degree.

### Limitations

This study has contributed to our understanding of values effects on contingency learning and links between perceived control and mood disturbance. However, there are limitations that require acknowledgment. We used the internet as a means of data collection. There were several reasons for choosing this form of data collection. Firstly, large samples were important to test hypotheses with adequate power. Moreover, internet data collection for larger samples is a cost effective and timely method. While these goals were achieved, a well acknowledged drawback of Internet data collection is high dropout rates. People can stop participating at any time in online experiments, and longer studies result in higher dropout levels (Hoerger, 2010). Whilst this is ethically desirable because, in contrast to a laboratory experiment, there is less social pressure to continue participation, reduced statistical power and possibly a biased final sample can result (Rogler, 1999; Pilkington and Msetfi, 2012). In this series of experiments, we anticipated high dropout rates due to the required duration of participation (the contingency judgment section of the experiment alone lasted a minimum of 5-min). However, despite this, the nature of the samples did not appear to have been compromised because data were consistent with patterns of learning reported from data collected for many years in the laboratory (e.g., Blanco et al., 2011, 2012).

## Conclusions

We set out to test whether values affect learning about control. Specifically, we tested whether values would affect illusory control and the relations between perceived control and symptoms. Both sets of hypotheses found partial support. There was evidence that values influenced the basic processes involved in contingency learning and perceived control via different modes of processing environmental or contextual stimuli. Associative learning theories that invoke elemental and configural processing can explain these findings. We also found that the perception of “being in control” when you are not, is not universally predictive of good psychological wellbeing and that the value ascribed to control is particularly important in relation to the euphoric symptoms of bipolar disorder. Thus researchers, and indeed clinicians, interested in perceived control, must acknowledge the part that values play in the complex relationship between perceived control and psychopathology.

### Conflict of interest statement

The authors declare that the research was conducted in the absence of any commercial or financial relationships that could be construed as a potential conflict of interest.
