# Supplementary material for: The relationship between mood state and perceived control in contingency learning: effects of individualist and collectivist values
Source: Front Psychol. 2015 Sep 29;6:1430. doi: 10.3389/fpsyg.2015.01430 (PMC4586436; doi:10.3389/fpsyg.2015.01430)
Supplement: Supplementary file 1 [file DataSheet1.PDF]

Appendix 1

Game testing room 1

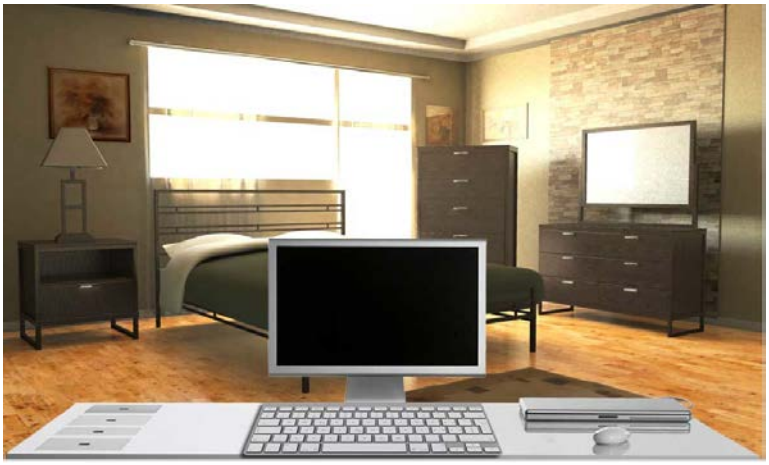

Game testing room 2

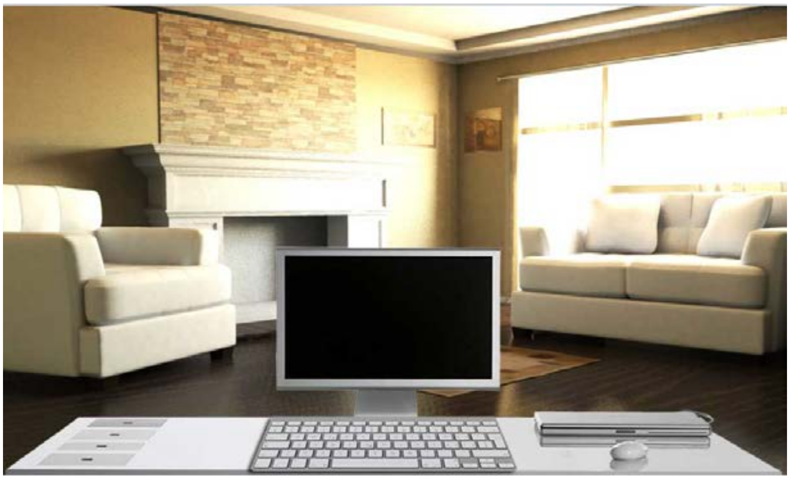

Appendix 2

Full instructions presented to participants for the contingency task

Screen 1

This is the final part of the study

We are working on developing a new online game. As part of the development process, we want to try to understand different levels of skill and control in the way people play all the different aspects of the game.

Start the test

Screen 2

Enter your details:
Please fill in your details in the following form. Your code is your initials followed by your date of birth. If your name is John Smith, born 10th September 1989, you should enter js100989
Code:
Age:
Gender: Male female

Submit your details and proceed

Screen 3

Browser / Sound / JavaScript Test Screen

Before we start, we must first check for compatibility in your browser.

Expected behaviour

In the shaded box directly below, you should be able to see a button marked "CLICK TO PLAY SOUND". Clicking on this button should cause a short sound file to be played.

If you are able to successfully play the sound clip, then scroll to the bottom of this page and click the button marked "CLICK TO PROCEED".

Unable to see the button / play sound?
The functionality in this test requires that **JavaScript** is enabled. Please ensure that you have not disabled JavaScript, and that it is not *blocked* through a browser plug-in.
The functionality in this test requires that the **Flash plug-in** is installed and enabled. Please ensure that you have not disabled Flash, and that it is not *blocked* through a browser plug-in (e.g. "FlashBlock").
If you are able to press the button, but cannot hear any sound, please check your sound volume.

Proceed to the Test
If you were able to successfully play the sound clip, then please click on the button below to proceed.

Screen 4

Instructions

We are working on developing a new online game. As part of the development process, we want to try to understand different levels of skill and control in the way people play all the different aspects of the game. In this particular game, which lasts around 5 minutes, your goal will be to try to make **a small ping sound** occur when you press the spacebar on the computer keyboard.

In order to play the game you will enter the **game testing room** where the computer has been set up. When you are in the **game testing room**, watch the **game** screen very carefully.

You will see a series of shapes and 3-letter computer codes presented one after the other on the TV screen.

If you see a triangle on the **game screen**, this is an **opportunity** to press the spacebar on the keyboard and see if you can make the ping sound occur. You should only press the space bar once during the time the triangle is on the screen. **However**, you should not press the spacebar every time you see the triangle. **Only press the spacebar some of the time** you see the triangle. Sometimes you might hear the ping sound when you have not pressed the spacebar.

Your skill over when and how you press the spacebar may give you control over the ping sound occurring. However, something about the game room, game console and game screen **could also** control **the ping sound**. So although you might play the game with a high level of skill, a fault in the wiring of the equipment, or the software itself could also have some control over the ping sound occurring. Or it might be the case that you have a lot of control because your high level of skill overrides any other issues and makes it possible for you to control the ping sound. It is also possible that the equipment and software are functioning perfectly but you simply have more or less control over the ping sound.

Therefore, when you are half way through the task and when you have finished, we will ask you to make a judgment about how much control you have over the ping sound occurring, and about how much control the other aspects of playing the game, like the game testing room, game console and game screen have.

If you are ready to play - click on the "Carry on" button!

Judgement screens

Judgement Screen 1: YOUR CONTROL

We would now like you to make some judgements about controlling the occurrence of the ping sound:

Your control and skill may have made it possible for you to control the occurrence of the ping.

If you had total control, and your pressing the space bar made the ping sound occur, click near the totally control end. If by pressing the spacebar, you totally prevented the ping sound from occurring, click near the totally prevent end.

Or it maybe that you had no influence over the ping sound occurring, click in the middle of the scale. Clicking nearer to the totally control end means MORE control, while clicking nearer to the totally prevent end means that you pressing the button interferes or prevents the ping from occurring to some degree. Clicking nearer to the middle, means less control.

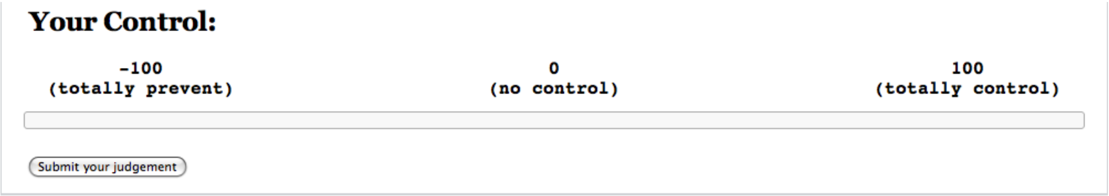

Judgement Screen 2: EXTERNAL CONTROL

Other things apart from your skill, such as the game testing room or the computer, **could also** control **the ping sound**. For example, there may be a fault in the wiring of the equipment, or a problem with the software itself that may have had some control over the ping sound occurring. It is possible that no such problems exist or that your skill could override them. How much control do you feel that these other factors had over the occurrence of the ping sound? Make your judgement by clicking in the scale below:

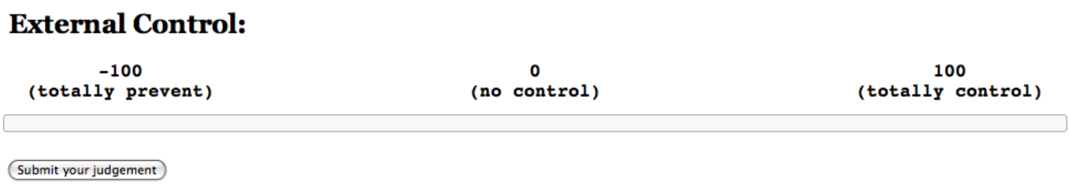

Thank You

Thank you for taking the time to participate in this study. If you have any further questions, you can contact the researcher on [email address] or visit our website at: [web address].

Appendix 3

Table A3.1

Pearson's *r* correlation coefficients for the relationships between subscales of the Individualism-Collectivism scale in Experiment 1.

Measure		HI	VI	HC	VC	Individualism
VI	*r*	.312**				
	p	<.001				
HC	*r*	.324**	**0.021**			
	p	<.001	**0.817**			
VC	*r*	.328**	0.16	.513**		
	p	<.001	0.073	<.001		
Individualism	*r*	.775**	.842**	.198*	.292**	
	p	<.001	<.001	0.026	0.001	
Collectivism	*r*	.374**	0.111	.843**	.894**	.286**
	p	<.001	0.214	<.001	<.001	0.001

NB: The correlation between VI and HC scores is shown in bold. Scores on these two subscales were used to assign people to culture groups. **≤ .001, *<.05

Table A3.2
Pearson's *r* correlation coefficients for the relationships between subscales of the Individualism-Collectivism scale in Experiment 2.

Measure		HI	VI	HC	VC	Individualism
VI	*r*	.244***				
	p	<.001				
HC	*r*	0.079	**-0.081**			
	p	0.157	**0.146**			
VC	*r*	.177***	.134*	.489**		
	p	0.001	0.016	<.001		
Individualism	*r*	.752**	.823**	-0.009	.195**	
	p	<.001	<.001	0.873	<.001	
Collectivism	*r*	.151**	0.036	.849**	.877**	.113*
	p	0.006	0.513	<.001	<.001	0.042

NB: The correlation between VI and HC scores is shown in bold. Scores on these two subscales were used to assign people to culture groups. ***≤ .001, **<.01, *<.05

Appendix 4
Priming Instructions

Priming Winning Values Instructions

We are working on developing a new online game. As part of the development process, we want to try to understand different levels of skill and control in the way that people play all the different aspects of the game, and why some people win at these games and some people do not. In this particular game, which lasts around 5 minutes, your goal will be to try to make a small ping sound occur when you press the spacebar on the computer keyboard**. As game developers we already know that, however much or little experience people have with online games, the level of control they have is really important when it comes to winning, and winning is important!**

In order to play the game you will enter the game testing room where the computer has been set up. When you are in the game testing room, watch the computer screen very carefully.

You will see a series of shapes and 3-letter computer codes presented one after the other on the computer screen.

If you see a triangle on the computer screen, this is an opportunity to press the spacebar on the keyboard and see if you can make the ping sound occur. You should only press the space bar once during the time the triangle is on the screen. However, you should not press the spacebar every time you see the triangle. Only press the spacebar some of the time you see the triangle. Sometimes you might hear the ping sound when you have not pressed the spacebar.

Your skill over when and how you press the spacebar may give you control over the ping sound occurring. However, something about the game room and computer could also control the ping sound. So although you might play the game with a high level of skill, a fault in the wiring of the equipment, or the software itself could also have some control over the ping sound occurring. Or it might be the case that you have a lot of control because your high level of skill overrides any other issues and makes it possible for you to control the ping sound. It is also possible that the equipment and software are functioning perfectly but you simply have more or less control over the ping sound.

Therefore, when you are half way through the task and when you have finished, we will ask you to make a judgement about how much control you have over the ping sound occurring, and about how much control the other aspects of playing the game, like the game testing room and computer have.

In computer games, most people like to win! Therefore you will also have the opportunity to win a prize for your performance on this task! At the end of the game we will ask you to answer a few more short questions about how you feel about taking part in the task, and then we will give you further information about whether you have won the competition!

If you are ready to play - click on the "Carry on" button!

Priming Participating Values Instructions

We are working on developing a new online game. As part of the development process, we want to try to understand different levels of skill and control in the way that people play all the different aspects of the game, and why some people win at these games and some people do not. In this particular game, which lasts around 5 minutes, your goal will be to try to make a small ping sound occur when you press the spacebar on the computer keyboard. **As game developers we already know that, however much or little experience people have with online games, the level of control they have is really important. Its not winning itself thats important, its how you play the game!**

In order to play the game you will enter the game testing room where the computer has been set up. When you are in the game testing room, watch the computer screen very carefully.

You will see a series of shapes and 3-letter computer codes presented one after the other on the computer screen.

If you see a triangle on the computer screen, this is an opportunity to press the spacebar on the keyboard and see if you can make the ping sound occur. You should only press the space bar once during the time the triangle is on the screen. However, you should not press the spacebar every time you see the triangle. Only press the spacebar some of the time you see the triangle. Sometimes you might hear the ping sound when you have not pressed the spacebar.

Your skill over when and how you press the spacebar may give you control over the ping sound occurring. However, something about the game room and computer could also control the ping sound. So although you might play the game with a high level of skill, a fault in the wiring of the equipment, or the software itself could also have some control over the ping sound occurring. Or it might be the case that you have a lot of control because your high level of skill overrides any other issues and makes it possible for you to control the ping sound. It is also possible that the equipment and software are functioning perfectly but you simply have more or less control over the ping sound.

Therefore, when you are half way through the task and when you have finished, we will ask you to make a judgement about how much control you have over the ping sound occurring, and about how much control the other aspects of playing the game, like the game testing room and computer have.

In computer games, some people like to win. However, we feel that the most important thing is the experience of playing the game! You will have the opportunity to win a prize for your performance on this task though. So at the end of the game we will ask you to answer a few more short questions about how you feel about taking part in the task, and then we will give you further information about whether you have won the competition.

If you are ready to play - click on the "Carry on" button!
